# Clinical and Urodynamic results of the Argus T^®^ sling in moderate and severe male stress urinary incontinence after radical prostatectomy – a 5 year prospective study.

**DOI:** 10.1590/S1677-5538.IBJU.2023.0003

**Published:** 2023-05-05

**Authors:** Adilson P. Carvalho, André B. Silva, Bruno R. Lebani, Eduardo R. Pinto, Mariana R. Felipe, Milton Skaf, Marcia E. Girotti, Stenio C. Zequi, Carlos A. R. Sacomani, Fernando G. Almeida

**Affiliations:** 1 Universidade Federal de São Paulo Departamento de Urologia São Paulo SP Brasil Departamento de Urologia, Universidade Federal de São Paulo, São Paulo, SP, Brasil; 2 Hospital do Câncer AC Camargo Serviço de Urologia São Paulo SP Brasil Serviço de Urologia Hospital do Câncer AC Camargo, São Paulo, SP, Brasil

**Keywords:** Urinary Incontinence, Stress, Suburethral Slings, Urodynamics

## Abstract

**Purpose:**

Sling as a therapeutic option for male stress urinary incontinence (SUI) has been reviewed in the last two decades, as it is a relatively simpliest surgery compared to artificial urinary sphincter and has the ability to modulate urethral compression. This study aims to evaluate the efficacy, rate of complications, quality of life and the effects on bladder emptying of the Argus T^®^ compressive and ajustable sling in moderate and severe male SUI treatment.

**Materials and Methods:**

Men eligible for stress urinary incontinence treatment after radical prostatectomy were recruited and prospectively evaluated, from March 2010 to November 2016. It was selected outpatient men with moderate and severe SUI, after 12 months of radical prostatectomy, who have failed conservative treatment. All patients had a complete clinical and urodynamic pre and post treatment evaluation, by means of clinical history, physical examination, urine culture, 1-hour pad test and ICIq-SF questionnaire. The UDS was performed after 12, 18 and 24 months postoperatively.

**Results:**

Thirty-seven men underwent sling surgery, 19 patients (51.4%) with moderate and 18 (48.6%) with severe SUI. The minimum follow-up time was 5 years. Overall, we had a success rate of 56.7% at 60 months follow-up. After surgery, we did not observe significant changes in the urodynamic parameters evaluated during the follow-up. No patient had urodynamic bladder outlet obstruction (BOO) after sling implantation. Readjustment of the Argus T^®^ sling was performed in 16 (41%) of the patients and 51% of the patients reported some adverse event.

**Conclusion:**

We demonstrate a long-term efficacy and safety of Sling Argus T^®^ as an alternative to moderate and severe male SUI treatment. Furthermore, in our study bulbar urethra compression does not lead to bladder outlet obstruction.

## INTRODUCTION

Urinary incontinence (UI) is a condition that drastically impacts patient’s quality of life (QoL), compromising physical, emotional, psychological, and social well being (1, 2). Stress urinary incontinence (SUI) is described as involuntary loss of urine concurrent with coughing, sneezing or physical effort. In men, SUI is usually associated with a history of prostate surgery, with an incidence of 2% to 10% after radical prostatectomy (RP), reaching 40% depending on surgeon’s experience or other risk factors (3–6).

Lifestyle changes, pelvic electrical stimulation and especially pelvic floor muscle training (PMT) are the most frequently recommended options for conservative treatment, associated with faster UI recovery after RP (7, 8). UI surgical treatment is recommended in patients with persistent SUI, estimating that 5-10% of patients will need surgical treatment after failure or incomplete result of conservative therapy (9).

The artificial urinary sphincter (AUS) is currently the gold standard for male UI treatment. Despite the good results, it is an expensive device with high mid and long-term revision rates (10-30%) (10). In addition, it requires patient’s skills to deactivate the device at the time of micturition. It is believed that the association of these factors may explain the search for treatments with new devices, including different types of slings.

In the last two decades, there has been a resurgence of interest in the use of slings as a therapeutic option for male UI, as it is a relatively simple surgery and has the ability to modulate urethral compression (11, 12). However, there are no studies in the literature that objectively demonstrate the presence and effects of an eventual urethral obstruction after sling implantation (1, 2, 13), with few studies having long-term follow-up (14–17).

Thus, given the hypothesis that Argus T may cause some degree of urethral obstruction, the present study aims to evaluate the efficacy, rate of complications, quality of life and the effects on bladder emptying of the Argus T^®^ Compressive Sling in the treatment of moderate and severe male SUI.

## MATERIALS AND METHODS

### Study population

**Figure 1:**
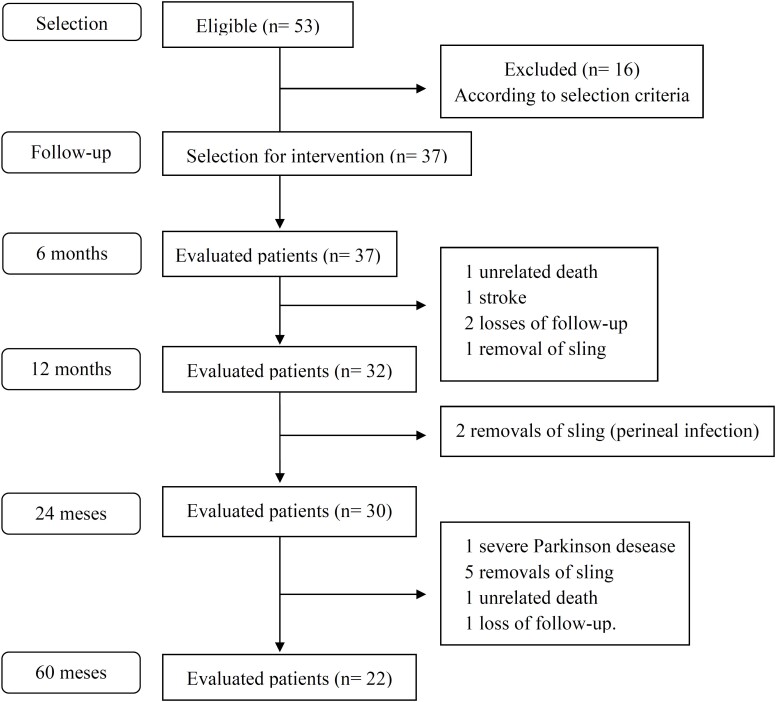
Study algorithm from patient’s selection to 5-year follow-up.

Men eligible for stress urinary incontinence treatment after radical prostatectomy were recruited and prospectively evaluated, from March 2010 to November 2016. The study protocol (Figure-1) was approved by the Research Ethics Committee, with free and informed consent signed by patients before inclusion in the study Pprotocol study number 1667/10.

It was selected outpatient men with moderate and severe SUI, 12 months after radical prostatectomy, who have failed to conservative treatment. Patients with mild SUI less than 12 months after prostatectomy, untreated urethral stricture, active urinary tract infection, patients undergoing radiotherapy, any geographic difficulties that could lead patient to abandon follow-up, psychiatric diseases, degenerative or demyelinating diseases, malnutrition, severe protein energy or terminal illness and patients who refused to participate the study were excluded.

### Study Assessment

SUI was objectively determined using 1-hour pad test, according to the protocol proposed by Abrams et al. (13). To perform the pad test, patients were instructed to drink half liter of water and place an absorbent previously weighed on a scale with an accuracy of 0.0001g. After 30 minutes, patients were instructed to walk, go up and down stairs and ramps for 10 minutes; sit down and get up from the chair 10 times; cough vigorously ten times; run for 1 minute; squat to pick up an object from the floor five times and wash hands under running water for 1 minute. After the end of 1-hour period, the absorbent was removed, weighed and the difference between values recorded.

According to this test, UI was classified as: grade 1 – test pad weight < 10 grams (g); grade 2 – pad test weight between 11 to 50 g, grade 3 – pad test weight between 51 to 100 g and grade 4 pad test weight > 100g (3). Patients were considered to have moderate loss when classified as grade 2 (11-50 g) and severe loss when classified as grade 3 and 4 (greater than 51 g).

All patients were evaluated at baseline through clinical history, physical examination, urine culture, 1-hour pad test, completion of the International Consultation on Incontinence Questionnaire-Short Form (ICIq-SF) (18) and urodynamic study (UDS). UDS was performed following the standards of good practices in urodynamics of the ICS (19), being analyzed the following variables: sensitivity, capacity, compliance, presence of detrusor hyperactivity and confirmation of the nature of the UI, Valsava Leak Pressure Point (VLPP). Parameters such as maximum urinary flow (Qmax), detrusor pressure at Qmax (Pdet@Qmax) and presence of post-voiding residue (PVR) were also evaluated for the presence of bladder outlet obstruction (BOO). For this purpose, the Bladder Outlet Obstruction Index (BOOI) was used, and patients with BOOI > 40 were considered obstructed, those with BOOI 20-40 were indeterminate, and those with BOOI < 20 were not obstructed (20).

### Surgical procedure

The sling Argus T^®^ (Promedon) device is composed by two columns and a silicone foam block with a total length of 45cm. A ring system (25mm and 15mm diameter) allows its fixation on the aponeurotic fascia of the obturator foramen musculature. The sling is implanted using two helical needles with a diameter of 3.5 mm, with a specific format to transpose the male pelvic obturator fossa.

After spinal anesthesia, the patient is placed in lithotomy with perineal shaving, with thorough assepsis and antisepsis, placement of sterile drapes and passage of a 16Fr indwelling urinary catheter with complete bladder emptying.

To implant the sling, we performed: 1) an infrascrotal longitudinal incision with dissection of the planes until identification of the bulbous and cavernous muscles, without violating the perineal fascia; 2) two small bilateral incisions in the inguinal crease 1.5 cm below the insertion of the adductor muscle, through which the TOT helical perforation needles passed, using the region of the ischiopubic bone, the cavernous muscles and the spongy bulb as a reference. The silicone block is then positioned around the bulbar urethra. Sling compression was adjusted based on retrograde urethral pressure of 35 cm of H2O, through a water column in a urethral probe positioned distally to the sling implant. After reaching the desired pressure, the device was fixed through the two washers: 3) perineal suture by layers.

The indwelling urinary catheter was removed after 12-24 hours, with hospital discharge after micturition. Antibiotic therapy was introduced 1 hour before the start of surgery and continued for 7 days (Ciprofloxacin 500mg every 12 hours). Sling readjustments were performed in an outpatient setting, under local anesthesia. In one of the inguinal folds, the washers were located and pulled to readjust the retrograde urethral pressure in 35 cm of H2O.

### Post operative evaluation

Patients were evaluated 1, 3, 6, 12, 18, 24 and 60 months after sling implantation, by means of clinical history, physical examination, urine culture, 1-hour pad test and ICIq-SF questionnaire. The UDS was performed after 12, 18 and 24 months postoperatively. The degree of postoperative continence was objectively evaluated at follow-up through the 1-hour pad test, with patients with a weight of up to 1g in the pad test being considered cured (dry) and patients with a reduction greater than 50% of the preoperative weight were considered improved. Success was considered as the sum of the two previous groups (improvement and cure) and failure in the other situations. Postoperative complications were grouped and stratified according to the Clavien-Dindo classification (21).

### Statistical Analysis

Data were summarized using mean and standard deviation for quantitative variables and absolute and relative frequency for categorical variables. The inferential analyzes used were the Student t test for independent samples and Mann-Whitney or Fisher’s exact test in the comparison of the moderate and severe groups, according to the characteristics of interest. Comparison of ICIq-SF score, Pad-test (g) and urodynamic test results from both groups were compared over time using ANOVA with repeated measures. Tukey method of multiple comparisons evaluated at which follow-up period there were significant differences, in addition to multiple comparisons by Bonferroni’s method. An alpha significance level of 5% was used. Statistical analyzes were performed using the statistical program R version 3.3.2.

## RESULTS

Between March 2010 and October 2011, among 53 patients eligible for the study, 37 met the eligibility criteria. The minimum follow-up time was 5 years. Out of the 37 men, 19 patients (51.4%) presented moderate, and 18 men (48.6%) had severe SUI. Demographic data of patients according to the subgroups are presented in Table-1.

**Table 1:** Demographics caracteristics according groups.

		Moderate	Severe	Total	p
		**N = 19**	**N = 18**	**N = 37**	
**Age (years)**		65.3 ± 7.1	66.1 ± 7.5	65.1 ± 7.2	0.742
**Time since prostate surgery (months)**		18.3 ± 4.5	19.1 ± 4.1	18.6 ± 4.7	0.835
**ICIQ SF (Score 0-35)**		16.5 ± 5.2	19.1 ± 3.9	17.8 ± 4.8	0.108
**Maximum bladder capacity (mL)**		359.1 ± 66.4	307.8 ± 113.6	336.1 ± 92.6	0.141
**Pdet.Qmax (cmH_2_O)**		23.3 ± 13.9	24.4 ± 14.8	23.8 ± 14.8	0.756
**Qmax. (mL/s)**		18.1 ± 9.6	14.8 ± 8.6	16.6 ± 9.2	0.356
**VLPP (cmH_2_O)**		84.1 ± 34	48.1 ± 38.6	68.6 ± 39.7	**0.015**
**Bladder sensibility (N)**					
	Normal	19	18	37	
	Augmented	0	0	0	
	Reduced	0	0	0	
**Hyperactivity (N)**					
	Yes	6 (31.6%)	4 (22.2%)	10 (27.0%)	0.714
	No	13 (68.4%)	14 (77.8%)	27 (73.0%)	
**Total**		**19 (100%)**	**18 (100%)**	**37 (100%)**	
**BOOI**		2.6 ± 17.4	-4.3 ± 24.3	-0.5 ± 20.9	0.475
**Post void residual (mL)**		16.3 ± 20.8	2.0 ± 7.2	9.9 ± 17.5	**0.025**
***Pad test* – 1 hour (g)**		18.6 ± 14.2	137.3 ± 68.4	78.0 ± 77.4	**<0.001**

ICIq-SF = International Consultation on Incontinence Questionnaire - Short Form; PdetQmax = Detrusor Pressure in the maximum flow; Qmax = Maximum flow; VLPP = Valsava Leak Pressure Point; BOOI = Bladder Outlet Obstruction Index

According to the inferential results, both groups presented the same profile regarding age (p=0.742), time from prostatectomy (p=0.835), ICIq-SF score (p=0.108), maximum bladder capacity (p=0.141), Pdet.Qmax (p=0.756), Qmax (p=0.356), presence of detrusor hyperactivity (p=0.714) and BOOI (p=0.475). The group with moderate SUI had higher Valsalva Leak Point Pressure (VLPP; p=0.015) and post-voiding residue (p=0.025), but lower 1-hour Pad test (p<0.001) when compared to their counterparts with more severe incontinence.

The ICIq-SF questionnaires and 1-hour pad test showed statistically significant variation over time (p<0.001), where multiple comparisons indicated a reduction in scores in all postoperative assessments (6, 12, 24 and 60 months) compared to the preoperative period (Table-2). Among the postoperative assessments, no significant differences were identified (Table-2).

**Table 2:** ICIq-SF,1 hour Pad test and Urodynamics variables during follow-up.

	Pre op	6 m	12 m	24 m	60 m	p value
**ICIq-SF**	17.8 ± 4.7	8.3 ± 7.4	9.0 ± 6.4	9.0 ± 6.4	8.1 ± 5.5	<0.001
**Pad-test (g)**	78.0 ± 77.4	16.2 ±31.7	11.3 ± 17.6	16.8 ± 29.5	5.7 ± 8.5	<0.001
**Maximum BladderCapacity (mL)**	336.1 ± 92.6	337.1 ± 81.3	327.0 ± 57.2	320 ± 61.4		0.104
**Qmax (mL/s)**	16.6 ± 9.2	14.8 ± 7.4	10.0 ± 3.2	11.0 ± 5.4		0.036
**PdetQmax (cmH _2_O)**	23.5 ± 14.1	29.9 ± 8.5	32.8 ± 12.5	39.3 ± 16.4		0.054
**Post void residual (mL)**	9.9 ± 17.5	18 ± 23.6	23.6 ± 50.2	17.8 ± 34.6		0.773

PdetQmax = Detrusor Pressure in the maximum flow; Qmax = Maximum flow; ICIq-SF = International Consultation on Incontinence Questionnaire - Short Form

After implantation of the sling, we did not observe significant changes in the urodynamic parameters evaluated during the follow-up (Table-2). There was a statistically significant reduction in Qmax at 12 and 24 months when compared to preoperatively. No patient had bladder outlet obstruction (BOO) after sling implantation.

**Figure 2:**
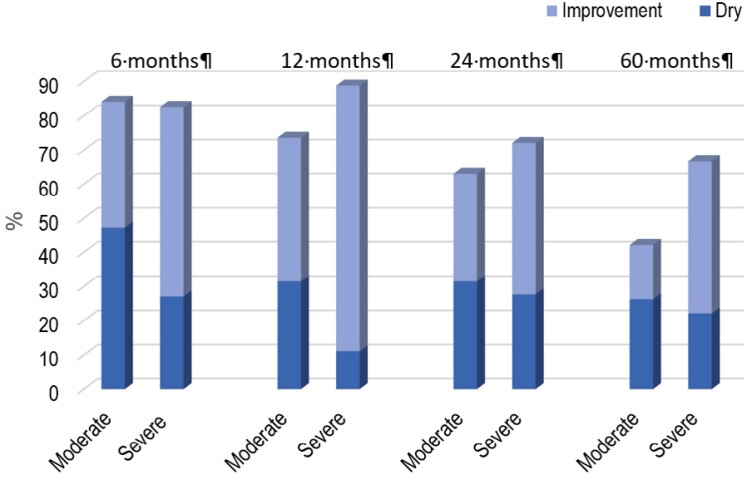
Clinical improvement and cure taxes during follow-up.

Considering the intention to treat, we observed that after 6, 12, 24 and 60 months, patients with moderate SUI were dry 47.3%, 31.5%, 31.5% and 26.3%; while 36.8%, 42%, 31.5% and 15.8% showed improvement and 84.2%, 73.7%, 63.1% and 42.1% were considered successful, respectively (Figure-2). In patients with severe SUI, we observed that after 6, 12, 24 and 60 months, 27.7%, 11.1%, 27.7% and 22.2% were dry, 55.5%, 77.7 %, 44.4% and 44.4% showed improvement, and 83.3%, 88.8%, 77.2% and 66.6% were considered successful.

Readjustment of the Argus T^®^ sling was performed in 16 (41%) of the patients: 9 (23%) in the first 6-month follow-up; 5 (12.8%) up to the 12-month follow-up; and 2 (5.1%) up to the 24-month follow-up; in two patients’ readjustment was necessary on two occasions. Readjustment was made only in those patients who had urinary leakage associated with significant bothering symptoms. In 8 patients it was necessary to remove the mesh, 3 of them in the first 2 years of follow-up and the others after 3 years of follow-up.

Preoperative variables age, ICIq-SF, VLPP, Qmax, Pdet.Qmax and post-void residual were tested to determine whether they would be related to success of surgery at the last available follow-up of 60 months. None of variables tested showed a relationship with the outcome of the surgery (age, p=0.558; ICIq-SF, p=0.088; VLPP, p=0.814; Qmax, p=0.525; Pdet.Qmax, p=0.219 and post-void residual, p=0.681), as shown in Table-3.

**Table 3:** Mean and standard deviation of pre op variables according to success and failure.

	Success	Failure	p value
**Age (years)**	66.2 ± 7.29	64.5 ± 7.16	0.558
**ICIq-SF**	18.6 ± 5.1	16.1 ± 2.8	0.088
**Qmax**	15.9 ± 8.4	14.1 ± 5.6	0.525
**PdetQmax**	21.9 ± 12.9	32.5 ± 17.3	0.219
**VLPP**	70.4 ± 43.8	66.9 ± 30.6	0.810
**Post void residual (mL)**	9.3 ± 17.8	6.7 ± 12.6	0.681

ICIq-SF = International Consultation on Incontinence Questionnaire - Short Form; PdetQmax = Detrusor Pressure in the maximum flow; Qmax = Maximum flow; VLPP = Valsava Leak Pressure Point

Fifty one percent of the patients reported some adverse event, and 30% of them had mild events that were resolved with medication or bladder catheterization. Sling removal was necessary in 8 (21%) cases: 2 due to infection that caused refractory perineal pain, 1 due to urethral fistula and 5 due to granulomas that evolved to extrusion of the distal silicon block. The main events observed in the study are shown in Table-4.

**Table 4:** Complications – Clavien-Dindo.

	Clavien – Dindo				
Complications	Grade I	Grade II	Grade III	Grade IV	Grade V
Low urinary tract infection	0	2	0	0	0
Acute urinary retention	0	0	5	0	0
Intense pain	4	0	0	0	0
Operatory wound infection	0	0	2	0	0
Uretral fistula	0	0	1	0	0
Granuloma	0	0	5	0	0

## DISCUSSION

Our study observed a significant success rate during the 60-month follow up, even in patients with severe urinary incontinence. There was a significant improvement in quality of life, which was maintained throughout the follow-up. In addition, we objectively proved, through the urodynamic study, that patients who underwent implantation of the Sling Argus T^®^ did not develop bladder outlet obstruction. Most complications were treated with drug therapy for a short period of time. This is the first clinical study using the Argus T^®^ Sling with long-term follow-up associated with post-operative urodynamic evaluation.

There are few studies in the literature addressing the treatment of male UI using the Argus T^®^ Sling that includes a prolonged follow-up period. In the present study, we performed a 60-month follow-up. Until then, the longest follow-up with Argus T^®^ sling described in the literature was performed by Castelejin et al, published in 2021 with a mean follow-up of 3.2 years (17). Previously, the largest reported follow-ups were those published by Romano et al., who followed 37 patients for 30 months (15), them Bauer et al. presented a case series with 42 patients, with a mean follow-up of 28 months (8) and a multicenter study published with 182 patients with a mean follow-up of 22 months (22).

We had an overall success rate of 56.7% at 60 months follow-up. Our mean term success rates were lower than those reported by Romano and Bauer in equivalent follow-up periods, but similar to the study conducted by Siracusano et al. (16), which included 182 patients and Castelejin et al. who achieved a 5-year total success rate of 55%. A reduction in success rates is expected over time, particularly in patients with moderate and severe SUI, related to implant extinction over time, recorded at later follow-up, as presented here.

Readjustment rate of compressive slings is high in published studies, ranging from 25 to 59% (14, 15, 23). In our study, readjustment occurred in 41% of patients, and readjustments were performed up to 24 months of follow-up, indicating stability after this accommodation period. In the 5-year follow up, we found a 51% complication rate. Despite of half patients have had some complication, most of them were treated conservatively. We had no Clavien IV-V complications. Out of the complicated patients, 34% were classified as Clavien III due to urinary retention, which in many studies is considered as Clavien I. Major complications requiring device removal occurs only in 21% of the patients. Our complications rate was similar to other reports. Castelejin NF, et al. in a multicenter study reported 64% overall complication rate (17). Hubner WA, et al. reported device removal in 16% of the patients in a 2-year follow up (23). Similar complications rates are seen in artificial urinary sphincter (AUS). Sacomani CAR, et al., in a median 5.2 years follow up after AUS implant, reported a necessity of device revision in 24 patients (19.8%), where 1 patient due to device malfunction, 5 patientes due to urethral atrophy, 3 patients due to persistent UI, and 15 patients due to urethral erosion with or without skin extrusion (24).

The ICIq-SF score presents a considerable correlation with the percentage of reduction of the 24-hour pad test, and when used during the pre and postoperative evaluation, it clearly captures the change in the severity of continence (16). In the present study, the preoperative assessment using the ICIq-SF showed a high score (mean of 17.8 points), compatible with the moderate/severe loss profile of the included patients. In the postoperative follow-up, there was a significant reduction in the indexes, up to 8.1 in 60 months, demonstrating a significant and stable reduction over 5 years. Similar values were described by Bauer et al. (8) and Horstmann et al. (25), despite being studies with shorter follow-ups.

We performed UDS pre and postoperatively (6 months, 12 months, and 24 months), in order to assess the impact that these devices, known as compressives, could have on the voiding pattern of our patients. There are few studies in the literature that objectively assess, through UDS, the development of an obstructive pattern in obstructive slings compared to non-compressive slings. The current justification for continence in non-compressive slings would be that continence would occur by repositioning the urethra and stretching the functional area of the urethra.

Horstmann et al. (25) evaluated 10 patients with UDS pre and postoperatively (3 and 6 months), after treatment of SUI with Advance^®^ sling. Likewise, Davis et al. (26) investigated 13 patients after implantation of the Advance^®^ sling by means of pre and postoperative (3 and 6 months) UDS, and in these series no evidence of obstruction was identified. Bauer et al., in a poster publication in 2011, evaluated 55 patients who underwent implantation of the Advance^®^ sling, through pre and postoperative UDS, 1-hour pad test and ICIq-SF questionnaire, indicating significant postoperative improvement. However, there was no change in maximum flow rates, detrusor pressure at maximum flow, micturition time and residual volume, that is, no postoperative obstructive pattern was identified. The success rate was 74% and the only urodynamic parameter that showed a significant difference was the VLPP in which the authors correlated this increase with the highest success rate (27).

On the other hand, with compressive slings the incontinence control is achieved through constant compression under the urethra, however, the compressive effects are not clearly described through urodynamic studies. In the present study, we clearly and objectively demonstrated that there is no bladder outlet obstruction with a sling implant, which is considered to be obstructive. In general, we did not prove statistically significant changes in urodynamic parameters. We believe that the compression caused by slings (both compressive and “non” compressive) seems to be sufficient to allow a degree of continence without necessarily causing obstruction at the time of urination.

Our study has limitations due to: the use of the 1-hour pad test to quantify SIU, since the 24-hour pad test is more detailed, although a recent systematic review has reported similar accuracy with great reproductibility (28); the loss of follow-up, although discreet, is another limitation, but inherent to the analysis profile and the long follow-up period; not having the standing cough test (29) to stratify patients, although the description of the test occurred later than the beginning of the study.

## CONCLUSIONS

Argus T^®^ Sling seems to have a long-term safety and efficacy in men with moderate and severe SUI. Furthermore, bulbar urethra compression seems to not lead to bladder outlet obstruction. Larger series are needed to confirm our findings.
